# Microbial therapeutics: An annotated selection of World Wide Web sites relevant to the topics in microbial biotechnology

**DOI:** 10.1111/1751-7915.12724

**Published:** 2017-04-25

**Authors:** Lawrence P. Wackett

**Affiliations:** ^1^ Department of Biochemistry, Molecular Biology & Biophysics BioTechnology InstituteUniversity of MinnesotaSt. PaulMN55108USA

## Microbial therapeutics review

### http://www.rle.mit.edu/sbg/wp-content/uploads/2013/02/Microbiome-therapeutics-Advances-and-challenges_2016_Advanced-Drug-Delivery-Reviews.pdf

This is a current review on microbial therapeutics. The review discusses additive and subtractive methods for modulating microbiota to improve health.

## BactoBots

### https://opentherapeutics.org/products/bactobots/

This web page describes a project supported by the National Science Foundation and Microbial Robotics to develop engineered bacteria to release therapeutic chemicals and kill cancer cells.

## Finch therapeutics

### http://finchtherapeutics.com

This is a commercial website for a company teaming up with other companies to develop microbial therapeutics. Projects include the treatment of inflammatory bowel disease and *Clostridium dificile* infections.

## Microbial ecosystem therapeutics

### https://www.ncbi.nlm.nih.gov/pubmed/23257018

This review article discusses options for replacing diseased gut microbiota with a healthy gut microbiota to restore health in human patients.

## Bacterial therapeutics

### http://www.highveld.com/microbiology/probiotics-therapeutics.html

This page highlights the book, *Probiotics and prebiotics: Current research and future trends*.

## Microbial communities as a source of therapeutics

### http://journal.frontiersin.org/researchtopic/4747/microbial-community-interactions-as-a-source-of-new-therapeutics

This Frontiers issue deals with microbial antibiotic resistance and efforts to identify new natural products and increasingly, alternative strategies for fighting microbial infectious disease.

## Using bugs as drugs

### http://www.phac-aspc.gc.ca/publicat/ccdr-rmtc/15vol41/dr-rm41s-5/innovation-eng.php

This review article discussed faecal microbial therapy and advocates for a more comprehensive treatment of modulating the broader intestinal ecosystem.

## Orally delivered microbial therapy

### http://www.mayoclinic.org/medical-professionals/clinical-updates/digestive-diseases/orally-delivered-microbial-therapy-has-potential-beyond-cdi

*Clostridium dificile* infections are being treated using faecal transplantation. This article discusses an alternative process of providing an oral pill containing spores that will pass into the intestine and modulate a healthy gut microbiota.

## Microbiome therapeutics programme

### http://www.serestherapeutics.com/our-science/microbiome-therapeutics-platform

This company provides services and products focused on analysing and treating diseased microbiomes, respectively.

## The Immuno‐Microbiome

### http://evelobio.com/#evelo-biosciences

Evelo Biosciences, working in collaboration with the Mayo Clinic, seeks to establish immuno‐microbiome cancer therapies.

## Microbial‐base therapy of cancer

### https://www.ncbi.nlm.nih.gov/pmc/articles/PMC3026423/

The use of bacteria to cause tumour regression has been investigated for decades, but recent developments in genomic sequencing and microbiome research have fuelled this area of research.

## Microbes meet cancer

### http://www.the-scientist.com/?articles.view/articleNo/45616/title/Microbes-Meet-Cancer/

This article discusses how the microbiome influences and enhances cancer chemotherapies, presumably through dealing with inflammation.

## Treating eczema with own microbiome

### http://www.sciencealert.com/scientists-are-making-personalised-eczema-treatments-from-people-s-own-microbes

The skin disorder eczema is thought to be due to an undesirable change in localized skin microbiota. In this context, a cream was made of healthy bacteria on skin to treat afflicted areas.

## Bacteria used for drug delivery

### http://www.wipo.int/wipo_magazine/en/2014/04/article_0002.html

This article highlights one companies approach in microbial therapeutics, largely from the perspective of intellectual property.

## Bacteria with thermostats delivering drugs

### http://www.futurity.org/engineered-bacteria-drug-delivery-1297492-2/

This news article discussed strategies for engineering bacteria to both thrive in the intestinal tract, as well as to clear when that is desirable.

## Therapeutic strategies for modulating gut inflammation

### http://www.nature.com/cti/journal/v5/n6/full/cti201638a.html

This is a broad‐based review on the effects of gut microbiota on modulating immune function.

## Bugs as drugs

### https://singularityhub.com/2015/11/22/bugs-as-drugs-seeking-microbial-cures-inside-body/

This news article provides an overview of modulating the human microbiome to deal with disease. The article focuses on some of the key researchers and emerging companies in the field.

